# Temperature-dependent properties of silver-poly(methylmethacrylate) nanocomposites synthesized by *in-situ* technique

**DOI:** 10.1186/1556-276X-9-42

**Published:** 2014-01-22

**Authors:** Noorsaiyyidah Darman Singho, Mohd Rafie Johan, Nurul Akmal Che Lah

**Affiliations:** 1 Nanomaterials Engineering Research Group, Advanced Materials Research Laboratory, Department of Mechanical Engineering, University of Malaya, Kuala Lumpur 50603, Malaysia

**Keywords:** Ag/PMMA nanocomposites, Surface plasmon resonance, Stability, Properties dependent temperature, 78.67.Sc, 73.20.Mf, 33.20.Ea, 07.85.Nc

## Abstract

Ag/PMMA nanocomposites were successfully synthesized by *in-situ* technique. Transmission electron microscopy (TEM) images show that the particles are spherical in shape and their sizes are dependent on temperature. The smallest particle achieved high stability as indicated from Zeta sizer analysis. The red shift of surface plasmon resonance (SPR) indicated the increases of particle sizes. X-ray diffraction (XRD) patterns exhibit a two-phase (crystalline and amorphous) structure of Ag/PMMA nanocomposites. The complexation of Ag/PMMA nanocomposites was confirmed using Raman spectroscopy. Fourier transform infrared spectroscopy spectra confirmed that the bonding was dominantly influenced by the PMMA and DMF solution. Finally, thermogravimetric analysis (TGA) results indicate that the total weight loss increases as the temperature increases.

## Background

Metal nanocomposites have attracted much attention due to their distinctive chemical and physical properties [1,2]. The properties of metal nanocomposites depend on the type of incorporated nanoparticles, their size and shape, their concentration, temperature, and interaction with polymer matrix. Silver (Ag) has been widely studied since it is more reactive than gold. However, appropriately stabilized Ag undergoes fast oxidation and easily aggregate in a solution. Among polymeric materials, poly(methyl methacrylate) (PMMA) was recognized as a polymeric glass with a wide range of applications. PMMA offers twofold advantages such as availability to carboxylate functional group for a chemical bonding with the metal ions and high solubility of PMMA in solvent-like dimethylformamide (DMF) for silver nitrate reduction. Therefore, Ag/PMMA nanocomposites are expected to be a hot spot area for its superior properties.

Earlier work on the synthesis of Ag/PMMA nanocomposites utilized sodium salt of acrylic acid via radiolysis method [3,4]. Deng et al. [5] has prepared Ag/PMMA nanocomposites by using PMMA and DMF via *in-situ* technique. They observed that the behavior of linear and nonlinear optical properties were different compared to the pure PMMA film. The main problem in polymer nanocomposites is to avoid the particles from aggregation. However, this problem can be solved by surface modification of the particles. This will improve the interfacial interaction between the metal particles and the polymer matrix.

In this paper, we used a simple procedure for the preparation of Ag/PMMA nanocomposites. In the first step, Ag nanoparticles were synthesized in water using the chemical reduction method [6-8]. This technique offers a systematic, efficient, and simple procedure for synthesis of Ag nanoparticles without decreasing the production rate. In the second step, Ag nanoparticles were mechanically mixed with PMMA dissolved in DMF to form nanocomposites at different temperatures. The temperature-dependent properties of nanocomposites were investigated by various techniques and their preparations of nanocomposites were discussed.

## Methods

Silver nitrate, AgNO_3_ (Thermo Fisher Scientific, Waltham, MA, USA) was selected as source of silver. Polyethylene glycol (PEG, MW 8000 in monomer units; Acros organics, Morris Plains, NJ, USA) was used as reducing agent. Daxad 19 (sodium salt of polynaphthalene sulfonate formaldehyde condensate, MW 8000; Canamara United Supply Company, Edmonton, AB, Canada) was used as stabilizer. N′N-dimethylformamide (DMF) (R & M Marketing, Essex, UK) used as solvent while PMMA (Acros Organics) as matrix. Four grams of AgNO_3_ was dissolved and stirred for 1 h in a mixture comprising of 100 mL distilled water, 4.5 g of PEG, and 5 g of Daxad 19 at 80°C. It was observed that the light brown solution transformed into a grey-black color, which indicates the formation of silver nanoparticles. The solution was then centrifuged at a maximum speed of 15,000 rpm, and washed with distilled water for several times [9]. Then, 10 g of PMMA was dissolved in 50 mL of DMF and mixed with 5 mL of silver nanoparticle solution at 80°C. The mixture was stirred for 1 h. This procedure was then repeated at 100°C and 120°C [10].

The physical shape and size of Ag/PMMA nanocomposites were observed by transmission electron microscopy (TEM; Leo Libra). The absorption spectrum was recorded by UV–VIS spectrophotometry (Cary Win UV 50, Agilent Technologies, Melbourne, Australia). The surface structure was characterized using Raman spectroscopy (Raman XploRA, Horiba, Kyoto, Japan) and Philips X'Pert MPD PW3040 X-ray diffraction (XRD; Amsterdam, The Netherlands) with CuKα radiation at 1.5406 Å. The zeta potential of Ag/PMMA nanocomposites was measured by Zetasizer (Zetasizer 3000HS, Malvern, Inc., Malvern, UK) while for thermogravimetry, TGA/SDTA 851 Mettler Toledo was used to measure the thermal properties. The Fourier transform infrared spectroscopy (FTIR) spectra were recorded on a spectroscopy (PerkinElmer, Spectrum 400, Waltham, MA, USA) within the range of 400 to 4,000 cm^-1^.

## Results and discussion

**Figure 1:**
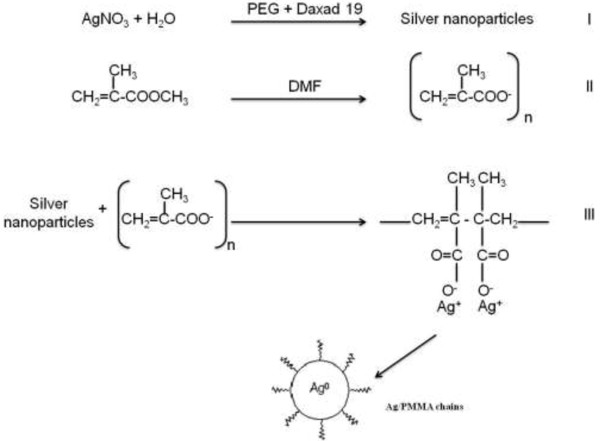
Mechanism of Ag/PMMA nanocomposites.

Figure 1 shows the proposed mechanism of Ag/PMMA nanocomposites. In Step 1, AgNO_3_ was dissolved in water to become Ag^+^ and NO_3_^-^. The color of the reaction solution changed slowly from colorless to light brown due to reduction of Ag^+^ to silver nanoparticles. In Step 2, PMMA was dissolved in DMF. As a result, the O-CH_3_ bond of MMA was dissociated, rendering very stable oxygen radical [11]. In step 3, silver nanoparticles were then dispersed in the MMA solution and coordinate to the oxygen atoms. This is a reasonable suggestion for the acrylate in PMMA because it is well suited for chemical bonding with the metal ions [12,13]. PMMA matrix prevents the aggregation of Ag nanoparticles and protects them through its carboxylate functional groups (Step 3).

**Figure 2:**
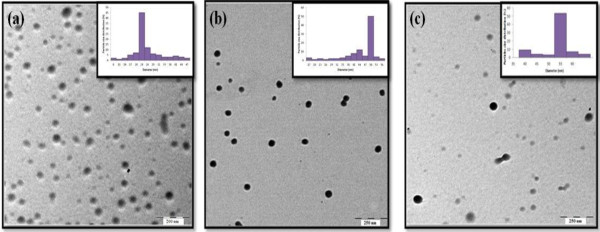
TEM images of Ag/PMMA nanocomposites synthesized at (a) 80°C, (b) 100°C, and (c) 120°C.

Figure 2 shows the TEM images of Ag/PMMA nanocomposites at different temperature. The particles are mostly in spherical shape. The smallest average particles size is 24 nm at 80°C. As the temperature increases, particle sizes increases up to 53 nm at 120°C. Ag/PMMA nanocomposites have narrow particle size distribution (inset) and highly dispersed at higher temperatures.

Table 1 shows the zeta potential and hydrodynamic diameters of the samples. It shows that the particles with smallest diameter have a more negative potential and much stable. The mutual repulsion among the particles sufficiently kept them separate and stabilizes the colloid at high negative potential. On the other hand, the low negative values of potential clearly indicate the instability of the aggregates.

**Table 1:** The zeta potential, thermal, and mass properties of Ag/PMMA nanocomposites synthesized at different temperatures

**Samples**	**Hydrodynamic diameter (nm)**	**Potential(mV)**	**Initial weight loss (%)**	**First decomposition weight loss (%)**	**Total weight loss (%)**	**Decomposition temperature (°C)**	**Stability temperature (°C)**
Pure PMMA	-	-	-	-	97.6	298	430
80°C	72	-61.0	3.7	75.9	79.6	253	409
100°C	96	-54.0	1.7	86.2	87.9	217	396
120°C	139	-35.1	20.4	71.4	91.8	207	370

**Figure 3:**
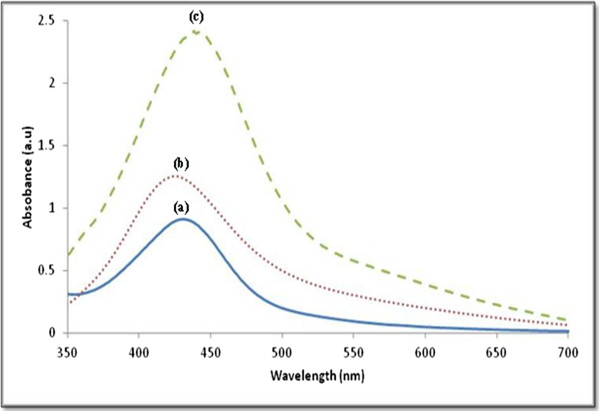
Absorption spectra for Ag/PMMA nanocomposites synthesized at (a) 80°C, (b) 100°C, and (c) 120°C.

Figure 3 shows the absorption spectra of all samples. The SPR bands are detected around 419 to 444 nm which indicated that the Ag/PMMA nanocomposites are in spherical shape. However, the red shift of SPR peaks as the temperature increases indicated the increase in particle size. These results are in good agreement with the TEM results (Figure 3).

**Figure 4:**
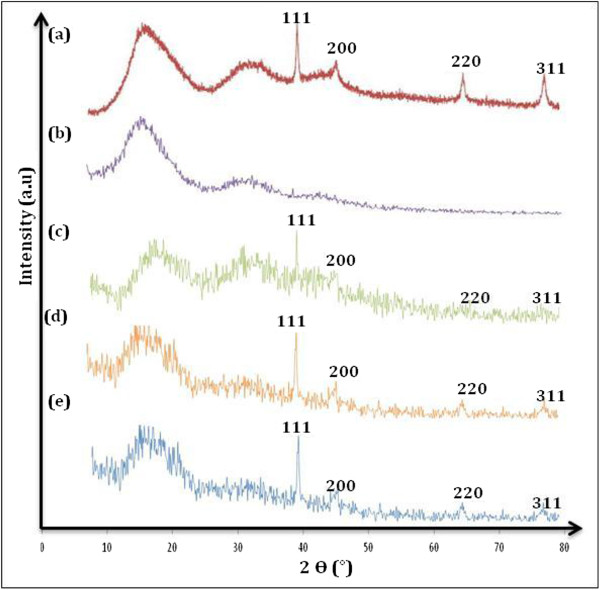
**XRD patterns (a,b) and nanocomposites at different temperatures (c,d,e). (a)** Ag nanoparticles and **(b)** pure PMMA. Temperatures: **(c)** 80°C, **(d)** 100°C, and **(e)** 120°C.

Figure 4 shows the XRD patterns for all samples at different reactant temperature. Figure 4a shows the XRD pattern of Ag nanoparticles. All the prominent peaks appeared at angle of 2*θ* = 38°, 44.44°, 64.54°, and 77.38° are corresponding to the (111), (200), (220), and (311) miller indices of face center cubic (fcc) of Ag. Figure 4b shows the XRD pattern for pure PMMA containing a broad peak at 19.62°. Meanwhile, Figure 4c,d,e shows the XRD pattern of Ag/PMMA nanocomposites at different reactant temperatures 80°C, 100°C, and 120°C which exhibits a two-phase (crystalline and amorphous) structure. The peak for (111) plane increases as the temperature increases up to 120°C. The Ag nanoparticles’ preferred alignment in PMMA is at the (111) plane. This can be explained from a viewpoint of thermodynamics since the preferred orientations of solid particles are known to be the perpendicular directions to the planes of lowest surface energy, which corresponds to the most densely packed planes for metallic materials [14,15].

**Figure 5:**
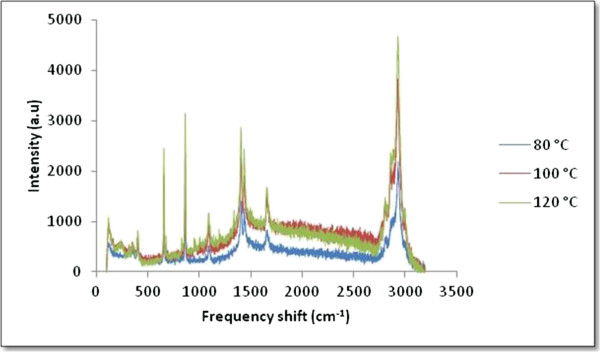
Raman spectra of Ag/PMMA nanocomposites synthesized at (a) 80°C, (b) 100°C, and (c) 120°C.

Figure 5 shows the Raman spectra of all samples. The band at approximately 240 cm^-1^ is due to the stretching vibration of Ag-N bond. Meanwhile, peaks at approximately 1,409 and 1,665 cm^-1^ can be attributed to symmetric and asymmetric C = O stretching vibrations, respectively [16]. Selective enhancement of these bands clearly indicates that C = O bonds of the carboxylate ions and Ag-N bond of the free amine groups are lying perpendicular to the surface of Ag nanoparticles. Notably, PMMA is a Raman-active compound with major bands at 600 cm^-1^ for (C-C-O) and (C-COO) stretch, 811 cm^-1^ for (C-O-C) stretch, 1,450 cm^-1^ for (C-H) in plane bending, and 1,728 cm^-1^ for (C = O) stretch [17]. The most prominent band appeared at 2,957 cm^-1^ is due to the C-H stretching vibration. The decreases of peak intensity at lower temperatures are due to the reduction of lattice vibration. The shape and size of the particles are strongly affected by the vibration; particles with the biggest size will allow the excitation of multipoles. As only the dipole transition leads to Raman scattering, the higher-order transitions will cause a decrease in the overall efficiency of the enhancement. Particles which are relatively smaller lose their electrical conductance [18].

**Figure 6:**
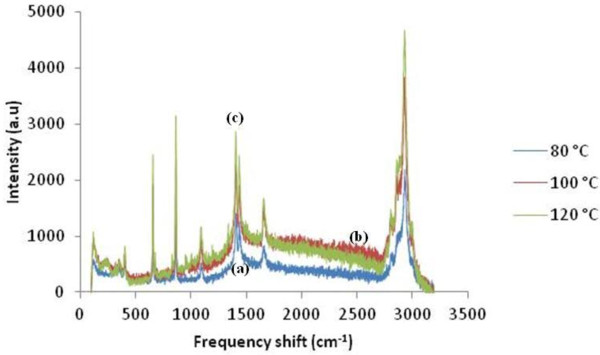
FTIR spectra for Ag/PMMA nanocomposites at (a) 80°C, (b) 100°C, and (c) 120°C.

Figure 6a,b,c shows the FTIR spectra of Ag/PMMA nanocomposites for 10% loading of Ag nanoparticles at 80°C, 100°C, and 120°C in the solution. The spectra showed that the bonding was dominantly influenced by the PMMA and DMF solution. This is due to the electrostatic attraction between acrylate ions of PMMA and Ag nanoparticles [19]. The main bands of DMF in Ag/PMMA nanocomposites spectra are clearly seen. The similarities between DMF and Ag/PMMA nanocomposite spectra verify the vital element of DMF in Ag/PMMA nanocomposites. It is found that the C = O (approximately 1,651 cm^-1^) and O = C-N-C (approximately 659 cm^-1^ vibration modes of DMF in Ag/PMMA nanocomposites was similar to those in DMF solvent. The bands correspond to C-O-C of the methoxy group, and skeletal C-C in Ag/PMMA nanocomposites appeared at 1,151 and 1,257 cm^-1^, respectively. These bands strongly affect their shape and size. A broad band of the carboxylic acid group due to the O-H (approximately 3,499 cm^-1^) in Ag/PMMA nanocomposites becomes broader as the temperature increases. The increase in water content may be originated from the environment or product of the chemical reactions. Both bands at approximately 1,065 and 1,088 cm^-1^ in Ag/PMMA nanocomposites are assigned to the sensitive metal complexes of methyl rocking vibrations coupled with a C-N vibration mode. The Ag/PMMA nanocomposite band at approximately 1,387 cm^-1^ is coupled in vibration, with the major contributions from CH_3_ deformation and C-N stretching mode. The interaction of the PMMA segments with Ag nanoparticles is demonstrated to be dependent on the regimes of the adsorption of polymer chain onto the surface.

**Figure 7:**
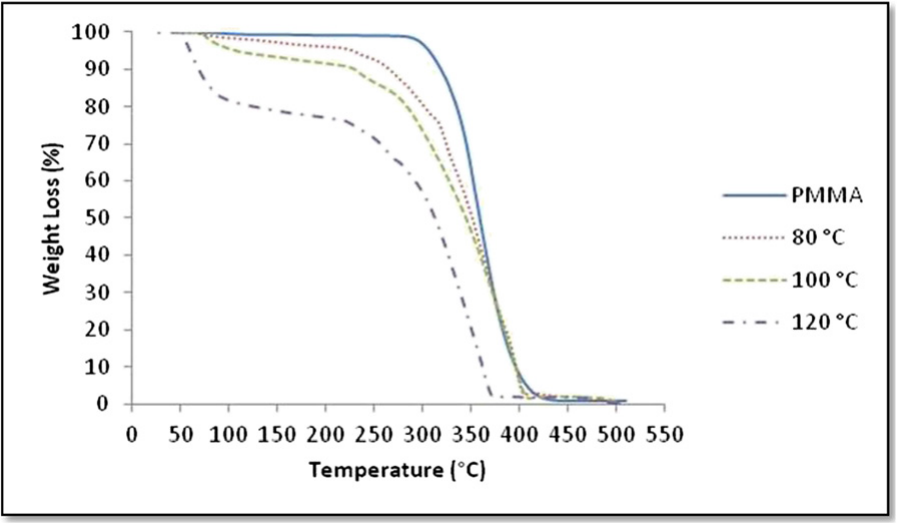
TGA curves of PMMA and Ag/PMMA nanocomposites synthesized at 80°C, 100°C, and 120°C.

Figure 7 shows the TGA curves of all samples. The first-stage decomposition started at about 253°C, 228°C, and 217°C for 80°C, 100°C, and 120°C, respectively. Table 1 summarizes the results. It is found that the maximum weight loss occurred for sample synthesized at 120°C with lower decomposition and stability temperature. This thermal stability can be ascribed to the fact that the presence of small amount of Ag in the polymer matrix confined the motion of polymer chains and served as a nucleation site for enhanced crystallization of nanocomposites [20,21]. It is evident that the Ag nanoparticles could efficiently improve the thermal stability of the composite in high temperature regions. The total weight loss percentage increases as the temperature increases. The incorporation of Ag nanoparticles shifted the decomposition toward higher temperatures. The observed behavior is most likely a consequence of the inhibiting effects of silver nanoparticles on some degradation stages of the thermo-oxidative degradation of PMMA.

## Conclusions

Ag/PMMA nanocomposites were successfully synthesized via *in-situ* technique. The size and distribution of Ag/PMMA nanocomposites were strongly dependent on the reactant temperatures. From the zeta potential analysis, the smallest particle has more negative potential and become much more stable. The red shifted and broader SPR bands were observed as the temperatures increases due to larger particle sizes. The peak for (111) plane in XRD results increases as the temperature increases up to 120°C with Ag nanoparticles preferred alignment in PMMA is at the (111) plane. From the Raman spectroscopy, we can conclude that the peak intensity decreases at the lower temperature due to the reduction of lattice vibration while from the FTIR spectra, the bonding was dominantly influenced by the PMMA and DMF solution due to the electrostatic attraction between acrylate ions of PMMA and Ag nanoparticles. TGA results showed that the total weight loss percentage increases as the temperature increases.

## Competing interests

The authors declare that they have no competing interests.

## Authors' contributions

MRJ conceived the idea and planned the experiments. NDS carried out the synthesis, characterization and analyzed the data. NACL carried out the TEM and analyzed the data. All the authors contributed to the preparation and revision of the manuscript, as well as, read and approved it.
